# IGFBP-2 acts as a tumour suppressor and plays a role in determining chemosensitivity in bladder cancer cells

**DOI:** 10.18632/oncotarget.27355

**Published:** 2019-12-17

**Authors:** Zhen Tang, David Gillatt, Edward Rowe, Anthony Koupparis, Jeff M.P. Holly, Claire M. Perks

**Affiliations:** ^1^ IGFs & Metabolic Endocrinology Group, Translational Health Sciences, Bristol Medical School, University of Bristol, Bristol BS10 5N, England; ^2^ Department of Surgery, Macquarie University Hospital, Macquarie University, Sydney, NSW 2109, Australia; ^3^ Department of Urology, Southmead Hospital and Bristol Urological Institute, Bristol BS10 5NB, England; ^*^ Co-senior authors

**Keywords:** IGFBP-2, methylation, chemosensitivity, invasion, bladder cancer

## Abstract

There are mixed reports on the role that IGFBP-2 plays in cancer progression, with some indicating a tumour suppressive role and others showing that IGFBP-2 may act as an oncogene. These apparent contradictions may be context and tissue specific. In this study we determined the role that IGFBP-2 played on the phenotype and chemosensitivity of a selection of bladder cancer cell lines and investigated how the abundance of IGFBP-2 was regulated. We found that IGFBP-2 was more abundant in the epithelial bladder cancer cells, RT4 and UMUC3 and absent in the more mesenchymal T24 and TCCSUP cells. Silencing IGFBP-2 using siRNA in epithelial RT4 cells promoted cell proliferation, invasion, colony formation, resulted in a reduction in epithelial (E-cadherin) and an increase in mesenchymal (N-cadherin) markers and increased sensitivity to cisplatin-induced cell death. Conversely, we observed the opposite effects when adding exogenous IGFBP-2 to the mesenchymal T24 cells. We determined that IGFBP-2 was epigenetically silenced via DNA methylation as the cells adopted a mesenchymal phenotype. Collectively these data suggest that IGFBP-2 acts as a tumour suppressor and marker of chemosensitivity in epithelial bladder cancer cells and that IGFBP-2 is epigenetically silenced by methylation to promote bladder cancer progression.

## INTRODUCTION

Epithelial-to-mesenchymal transition (EMT) describes the process by which cells change from an adherent epithelial into a highly motile mesenchymal-like cell. EMT is characterised by alterations in the abundance of epithelial cell surface markers, such as a reduction in E-cadherin [1] and the acquisition of mesench-ymal proteins, such as N-cadherin [2]. The loss of epithelial features and a gain of mesenchymal characteristics is associated with increases in cell proliferation, migration and invasion, and an increase in colony formation [3–5].

IGFBP-2 acts in both IGF-dependent and -independent ways. In the presence of IGFs, IGFBP-2 reduces the effects of IGFs by sequestering free bioactive IGFs into inactive IGF:IGFBP-2 complexes [6], and the intrinsic IGF-independent effects, that have been demonstrated in many cancers occurs by binding, via their RGD sequence, to integrin receptors, similar to that reported in prostate [7]. The IGFs are widespread regulators of most cell functions; their interactions with IGFBPs can reduce the clearance of IGFs, sequester IGFs in the extracellular matrix or on cell surfaces, via interactions with proteoglycans, and hence enhance targeting of the IGFs to specific sites of action and also constrain receptor binding by competition. The different effects of IGFBPs depend on interactions with extracellular matrix, cell surfaces and extracellular proteases; all of which can vary in a tissue specific manner and therefore confer specificity, depending on context, to the multiple potential IGF actions [8].

There are mixed reports on the role of insulin-like growth factor binding protein-2 (IGFBP-2) in cancer progression. Some studies report that it acts as an oncogene as it is commonly expressed in high grade tumours and promotes cancer development and progression in a number of cancers, including those of the breast [9–11], prostate [7, 12] and brain [13, 14]. However, others report that aggressive cancers have little or no expression of IGFBP-2 compared with more differentiated forms of the tumours. For example, increased levels of IGFBP-2 are found in rapidly growing non-invasive brain tumours, whereas low/undetectable levels are observed in malignant invasive brain tumours [15].

There are limited reports on the role of IGFBP-2 in bladder cancer progression. In 2005, it was reported that over-expressing IGFBP-2 in KoTCC-1 cells (established from the ascites of a 23-year-old woman with peritonitis carcinomatosa) that possess little IGFBP-2, caused the cells to become more proliferative and motile [16]. The same group also determined that IGFBP-2 mRNA expression levels and the relative expression ratio of IGFBP-2 to IGFBP-3 mRNAs in 97 bladder cancer specimens significantly correlated with pathological stage, lymph node metastasis and vascular invasion [16]. These data suggest that IGFBP-2 may play a role in promoting progression of bladder cancer.

The apparent contradictions in the role of IGFBP-2 in relation to its expression may be explained by the different ways in which IGFBP-2 can be regulated, that will be specific to the context of a tissue-type. For example, the levels and function of IGFBP-2 can be regulated by promoter methylation, hormones and proteases (see review [17]).

In this study we determined the role that IGFBP-2 played on proliferation, invasion, EMT, colony formation and chemosensitivity in a selection of bladder cancer cell lines and investigated how the abundance of IGFBP-2 was regulated.

## RESULTS

We initially assessed the levels of IGFBP-2 in relation to the epithelial or mesenchymal characteristics of each of the cell lines. RT4 cells are the most epithelial-like with high levels of E-cadherin and no N-cadherin; these cells exhibited the highest levels of IGFBP-2. T24 cells were the most mesenchymal-like demonstrating the highest levels of N-cadherin with no E-cadherin: we observed that this cell line also had undetectable levels of IGFBP-2. Similarly, TCCSUP, another mesenchymal cell line, has low levels of N-cadherin, no E-cadherin and low levels of IGFBP-2. UMUC3 is classified as epithelial-like despite having negligible levels of E-cadherin, however, it did produce relatively small amounts of IGFBP-2 and had no N-cadherin (see Supplementary Figure 1A).

### Effect of silencing IGFBP-2 in epithelial RT4 cells on the growth, invasion, colony formation and abundance of EMT markers

**Figure 1:**
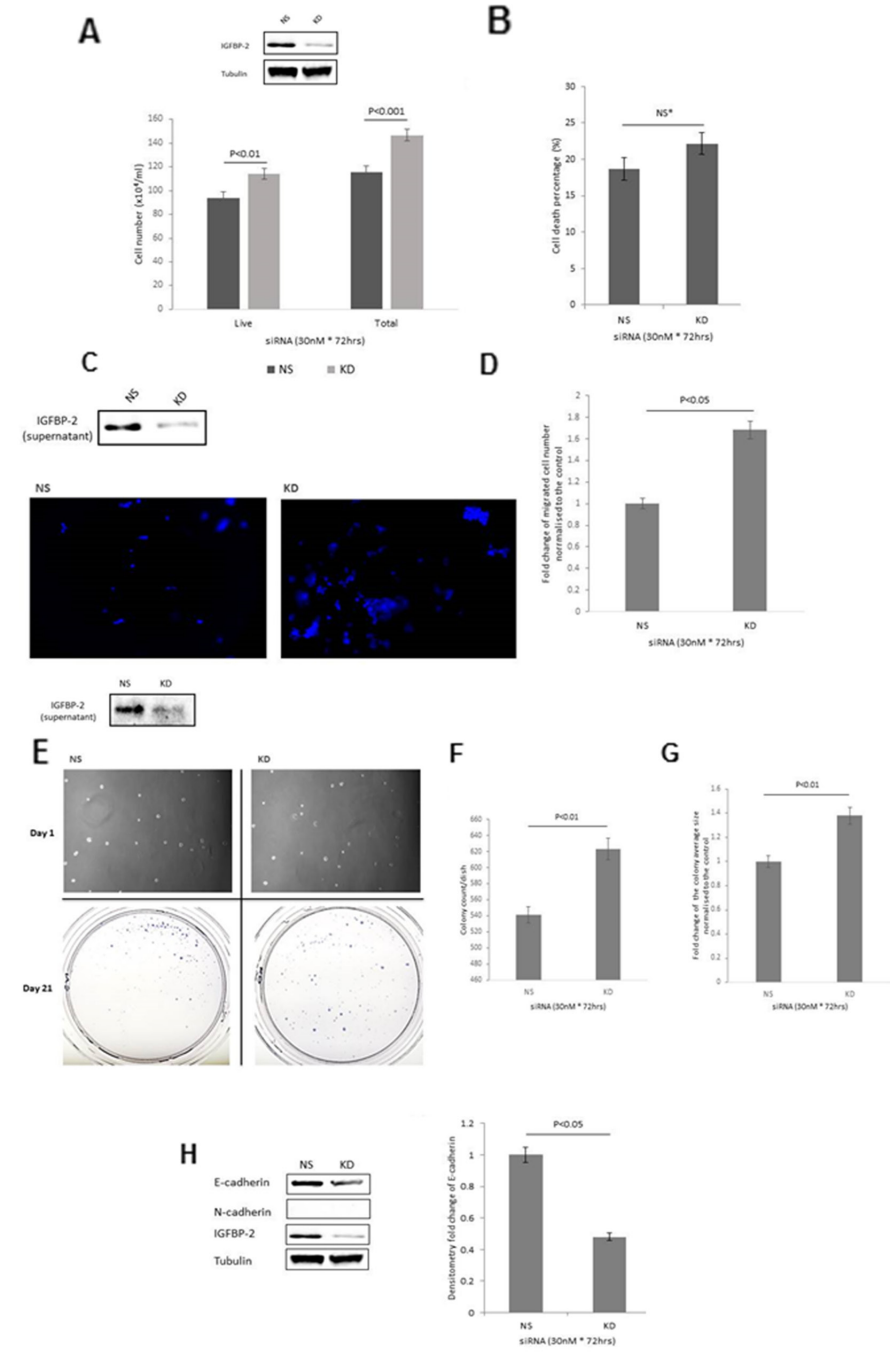
Shows the effect of IGFBP-2 silencing in RT4 cells on **(A)** total cell number **(B)** cell death **(C & D)** invasion (image shows invaded cells with the nuclei stained with DAPI (in blue); X 10 magification) and graph indicates the mean change in number of invaded cells **(E)** colony formation; images of cells on day 1 and of colonies on day 21; x 10 magnification. **(F&G)** graphs represent the change in colony count and fold change of the average colony size respectively. **(H)** EMT markers, E- and N-cadherin and the graph shows the mean optical density change in E-cadherin. Inserts in A, C, E & H show a Western blot indicating effective IGFBP-2 silencing. Graphs show the mean and SEM of data from 3 separate experiments each performed in triplicate. Data were analysed with SPSS 12.0.1 for Windows using one-way ANOVA followed by least significant difference (LSD) post-hoc test. A statistically significant difference was present at p<0.05. NS= non-silencing control, KD= knockdown with siRNA.

With IGFBP-2 silenced using siRNA an increase in both total cell number (by 27.2% (p<0.001)) and live cell number (by 21.6% (p<0.01)) was observed (Figure 1A). There were no significant changes in the level of cell death between the two groups (Figure 1B). Effective silencing of IGFBP-2 is indicated in Figure 1A insert. A 68% increase (p<0.05) in cell invasion (Figure 1C&D) was detected. Effective silencing of IGFBP-2 is indicated in Figure 1C insert. The cells with IGFBP-2 silenced formed more and larger colonies: a 15% increase in colony forming efficiency (CFE) (p<0.01) (Figure 1E&F) with a 1.38 fold increase in the average size of each colony (p<0.01) (Figure 1E&G). Effective silencing of IGFBP-2 is indicated in Figure 1E insert. These phenotypic changes in response to silencing IGFBP-2 (Figure 1H insert) were associated with a marked reduction in the epithelial marker E-cadherin (p<0.05) but no apparent induction of N-cadherin (Figure 1H).

### Effect of adding exogenous IGFBP-2 to mesenchymal T24 and TCCSUP cells on the growth, invasion, colony formation and abundance of EMT markers

**Figure 2:**
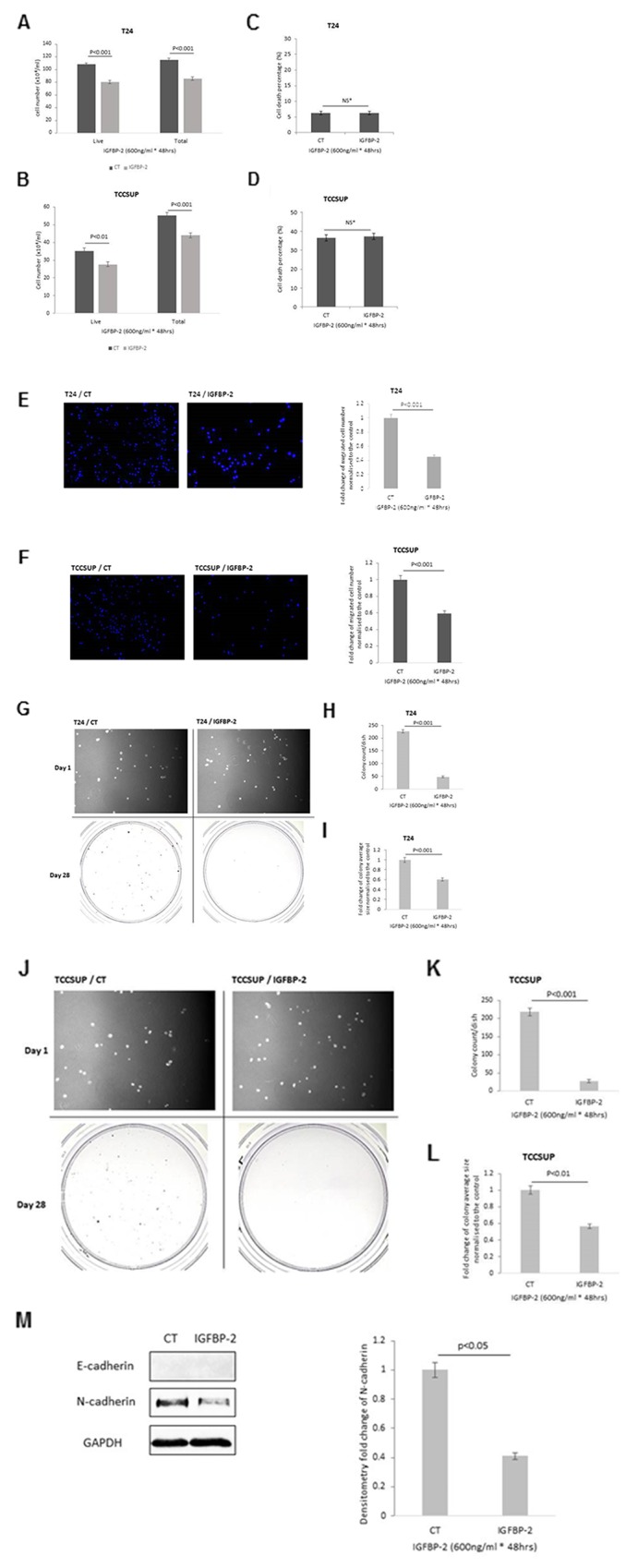
Shows the effects of adding exogenous IGFBP-2 (600ng/ml) to T24 and TCCSUP cells on **(A & B)** total cell number respectively, **(C & D)** cell death respectively **(E & F)** invasion (images show invaded cells with the nuclei stained with DAPI (in blue); X 10 magification and graph indicates the mean change in number of invaded cells **(G, H & I)** colony formation for T24 cells; images of cells on day 1 and of colonies on day 28; x 10 magnification. Graphs represent the change in colony count and fold change of the average colony size. Graphs show the mean and SEM of data from 3 separate experiments each performed in triplicate. Data were analysed with SPSS 12.0.1 for Windows using one-way ANOVA followed by least significant difference (LSD) post-hoc test. A statistically significant difference was present at p<0.05. CT=control. **(J-L)** show the effects of adding exogenous IGFBP-2 (600 ng/ml) to TCCSUP cells on colony formation; images of cells on day 1 and of colonies on day 28; x 10 magnification. Graphs represent the change in colony count and fold change of the average colony size and (M) EMT markers, E- and N-cadherin and the graph shows the mean optical density change in N-cadherin. Graphs show the mean and SEM of data from 3 separate experiments each performed in triplicate. Data were analysed with SPSS 12.0.1 for Windows using one-way ANOVA followed by least significant difference (LSD) post-hoc test. A statistically significant difference was present at p<0.05. CT=control.

T24 and TCCSUP cells were treated with an effective anti-proliferative dose of IGFBP-2 (600 ng/ml) determined from dose response curves (Supplementary Figure 1B). Exogenous addition of IGFBP-2 to T24 and TCCSUP cells showed a 25.8% and 20.2% decrease in total cell number respectively (p<0.001 for each) and a 25.9% and 21.1 % reduction in live cell number (p<0.001 & p<0.005) respectively (Figure 2A&B) with no change in cell death (Figure 2C&D). Exogenous IGFBP-2 also induced a 55% and 41% decrease in cell invasion in T24 (Figure 2E) and TCCSUP cells (Figure 2F) respectively (p<0.001 for each). A reduction in colony formation of 78.8% (p<0.001) (Figure 2G&H) and of 87.6% (p<0.001) (Figure 2J&K) was found in IGFBP-2-treated T24 and TCCSUP cells, respectively. Similarly, the fold decrease in average size of each colony were 0.60 (p<0.001) (Figure 2G&I) and 0.57 (p<0.01) (Figure 2H&L) respectively. The phenotypic changes in response to the exogenous addition of IGFBP-2 in T24 cells were accompanied by a 59% (p<0.05) reduction in the abundance of N-cadherin with no detectable induction of E-cadherin (Figure 2M). Effects of IGFBP-2 on N-cadherin were not assessed in the TCCSUP cell line as levels of N-cadherin are relatively low already.

### To determine if the phenotypic effects of IGFBP-2 are independent of IGF-I

**Figure 3:**
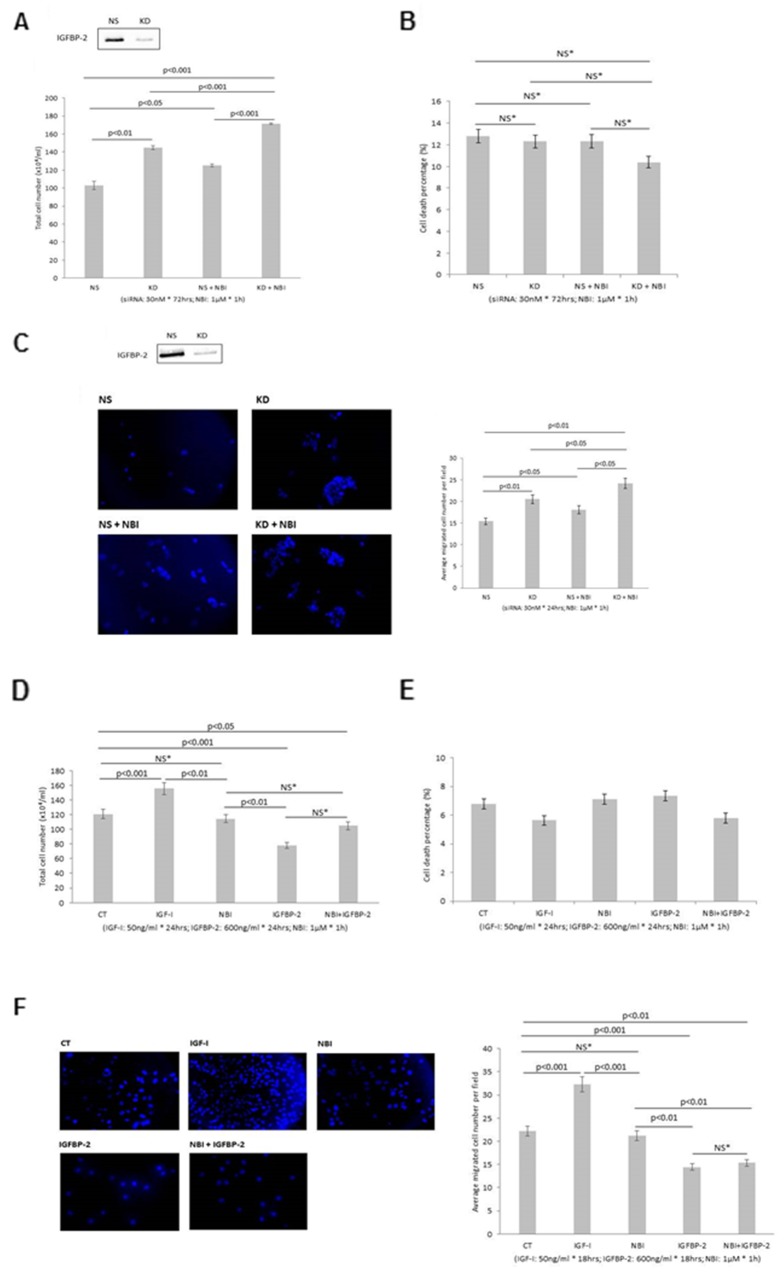
Shows the effects of manipulating IGFBP-2 in RT4 and T24 cells in the presence of NBI-31772 (1µM) **(A, B & C)** Show the effect of silencing IGFBP-2 in the presence or absence of NBI-31772 (NBI) in RT4 cells on total cell number, cell death and invasion respectively. **(D, E & F)** Show the effect of adding exogenous IGFBP-2 in the presence or absence of NBI-31772 in T24 cells on proliferation, cell death and invasion respectively. Inserts in A & C show a Western blot indicating effective IGFBP-2 silencing. Invasion images show invaded cells with the nuclei stained with DAPI (in blue); X 10 magification and the corresponding graph indicates the mean change in number of invaded cells. Graphs show the mean and SEM of data from 3 separate experiments each performed in triplicate. Data were analysed with SPSS 12.0.1 for Windows using one-way ANOVA followed by least significant difference (LSD) post-hoc test. A statistically significant difference was present at p<0.05.

Figure 3A shows that with RT4 cells pre-treatment with NBI-31772 alone led to a significant increase in cell growth (21.7% p<0.05), suggesting that IGFs had been released from IGFBPs, predominantly IGFBP-2 as this is the main one secreted by these cells, and were able to increase cell growth. This indicates that IGFBPs, predominantly IGFBP-2, acts at least partly by sequestering IGFs since when IGFs are displaced from the IGFBPs this stimulates cell growth and silencing IGFBP-2, when it can no longer sequester IGFs also results in cell growth. Silencing IGFBP-2, as we observed previously, caused an increase in cell growth (by 40.9%) of RT4 cells. In the presence of NBI-31772, silencing IGFBP-2 still induced a comparable increase in cell growth which was additive to the effects of NBI-31772 alone on cell growth (66.3%, p<0.001). In the presence of NBI-31772, when any interaction of IGFBP-2 with IGFs is prevented, the observation that silencing IGFBP-2 still results in cell growth indicates that the presence of IGFBP-2 can inhibit cell growth independent of any interaction with IGFs. In combination these results suggest that the effects of IGFBP-2 on these cells are both IGF-dependent and -independent. There were no effects on the levels of cell death (Figure 3B; effective silencing of IGFBP-2 shown in Figure 3A insert). We confirmed that exogenously added IGF-I was able to dose-dependently increase cell proliferation in the RT4 cells (Supplementary Figure 1D).

With RT4 cells, silencing IGFBP-2 alone increased cell invasion (by 33.1%) and in the presence of NBI-31772, silencing IGFBP-2 still induced a comparable increase in invasion which was additive to the effects of NBI-31772 alone on cell invasion growth (56.9% <0.01), suggesting again both IGF-dependent and -independent effects of IGFBP-2 on invasion in RT4 cells (Figure 3C-effective silencing of IGFBP-2 shown in insert). We confirmed that exogenously added IGF-I was able to increase cell invasion in the RT4 cells (Supplementary Figure 1E&F).

Figure 3D shows that with T24 cells, exogenously added IGF-I induced a significant increase in total cell number (28.6%, p<0.001) while NBI-31772 did not, suggesting that no appreciable IGF was released from any IGFBPs present. Exogenous IGFBP-2 led to a decrease in cell growth (35.3%, p<0.001), and this effect was negated in the presence of NBI-31772, suggesting that exogenously added IGFBP-2 reduces cell growth through sequestering endogenous IGFs and can no longer do this in the presence of NBI-31772: this implies an IGF-dependent action of the exogenously added IGFBP-2 with these cells. There were no effects on the levels of cell death (Figure 3E).

With T24 cells, IGF-I induced a significant increase in cell invasion (45.5%, p<0.001) while NBI-31772 did not. Exogenous IGFBP-2 treatment led to a decrease in cell invasion (35%, p<0.001), and this was unaffected by the presence of NBI-31772 suggesting that the effects of IGFBP-2 on cell invasion were not blocked by NBI-31772 and were therefore independent of IGFs (Figure 3F).

### Assessment of epigenetic modification of IGFBP-2 in bladder cancer cells

**Figure 4:**
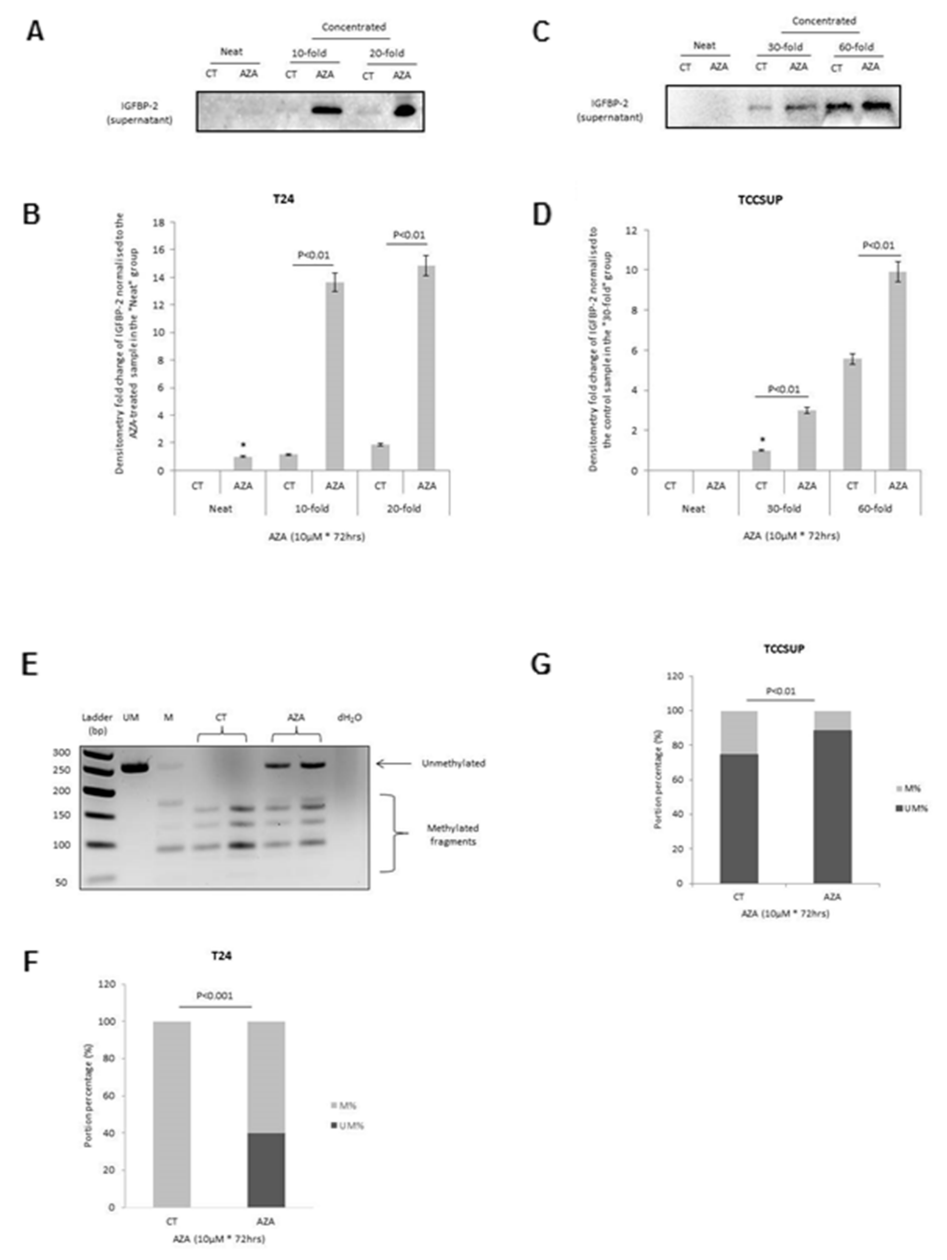
Effect of 5-AZA on the abundance and methylation status of the *IGFBP-2* gene promoter **(A & B)** show a Western blot of IGFBP-2 in the cell supernatant (neat, 10 and 20-fold concentrated) and a graph showing fold change in abundance after treatment with 5-AZA (10µM; 72 hrs) in T24 cells. **(C & D)** show the same as in A & B for TCCSUP cells. **(E)** shows a representative gel indicating methylated (M) and unmethylated (UM) bands representing IGFBP-2 following 5-AZA treatment of T24 cells and this is represented as % M and UM in the graph in **(F)**. **(G)** shows a graphical representation of % M and UM bands representing IGFBP-2 following 5-AZA treatment of TCCSUP cells. Gels and graphs are representative of experiments repeated at least three times. Graphs show the mean and SEM. Data were analysed with SPSS 12.0.1 for Windows using one-way ANOVA followed by least significant difference (LSD) post-hoc test. A statistically significant difference was present at p<0.05.

To investigate whether the loss of IGFBP-2 in mesenchymal-like bladder cancer cells potentially resulted from epigenetic regulation, we initially treated the mesenchymal T24 and TCCSUP cells with a demethylating agent, AZA. We observed a clear increase in the abundance of IGFBP-2 following AZA-exposure in the T24 cells (Figure 4A&B) and this was less marked in the TCCSUP cells. Having shown that IGFBP-2 was re-expressed in T24 and TCCSUP cells following AZA treatment, COBRA was then performed with these two cell lines to assess any alterations in gene methylation status of the *IGFBP-2* promoter and to confirm whether the loss of IGFBP-2 in mesenchymal-like bladder cancer cell lines could be the result of an epigenetic change. With T24 cells, the promoter region of the *IGFBP-2* gene was completely methylated in the control samples, and the treatment with AZA led to the demethylation of this gene with a significant increase in the percentage of unmethylated DNA bands from 0 (in control cells) to 39.9% (in AZA-treated cells) (p<0.001) (Figure 4E&F). With TCCSUP cells, very low levels of methylation were observed in the control cells. However, gene demethylation, but to a much smaller extent than observed in T24 cells, was detected in TCCSUP samples upon AZA treatment, with the percentage of unmethylated DNA bands increasing from 74.8% (in control cells) to 88.6% (in AZA-treated cells) (p<0.01) (Figure 4G).

### AZA mimics the phenotypic effects and the alterations in EMT markers observed in the presence of exogenous IGFBP-2 in T24 mesenchymal-like bladder cancer cells

**Figure 5:**
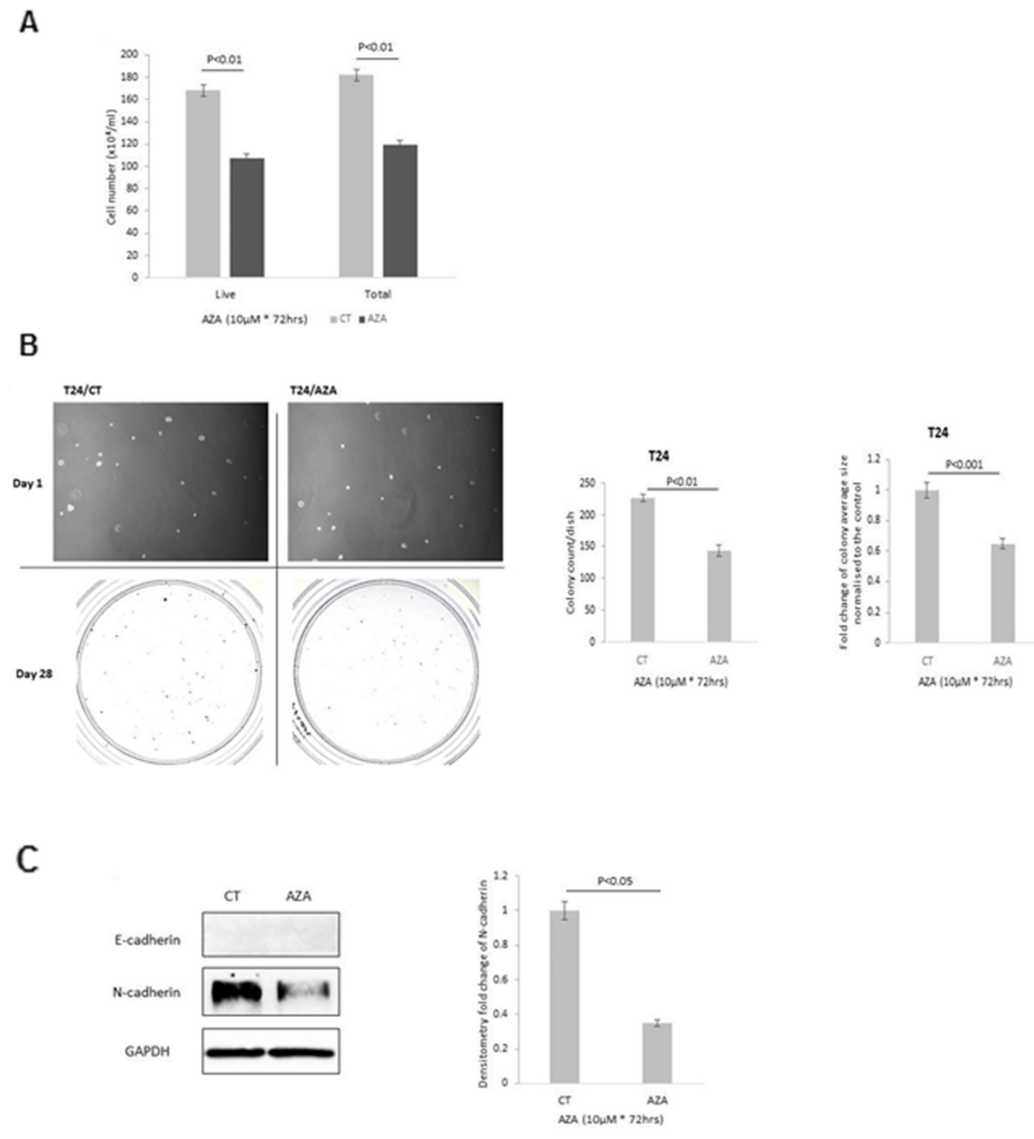
Effect of 5-AZA on T24 cells with respect to **(A)** cell growth **(B)** colony formation; images of cells on day 1 and of colonies on day 28; x 10 magnification. Graphs represent the change in colony count and fold change of the average colony size respectively. **(C)** EMT markers, E- and N-cadherin and the graph shows the mean optical density change in N-cadherin. Graphs show the mean and SEM of data from 3 separate experiments each performed in triplicate. Data were analysed with SPSS 12.0.1 for Windows using one-way ANOVA followed by least significant difference (LSD) post-hoc test. A statistically significant difference was present at p<0.05.

As a clear effect on the methylation of IGFBP-2 following AZA treatment was observed in the T24 cells, we assessed if AZA mimicked the phenotypic effects of adding exogenous IGFBP-2. AZA decreased both total cell number (by 34.3%, p<0.001) and live cell number (by 36.4%, p<0.001) (Figure 5A). With T24 cells colony forming efficiency (CFE) decreased by 36.7% (p<0.01) and the average size of each colony showed a 0.6 fold decrease (p<0.001) relative to control cells (Figure 5B). With the treatment of AZA, the abundance of N-cadherin was reduced by 65% (p<0.05) with no observed changes in E-cadherin (Figure 5C).

### The presence of IGFBP-2 in tumours may affect the response to chemotherapy

**Figure 6:**
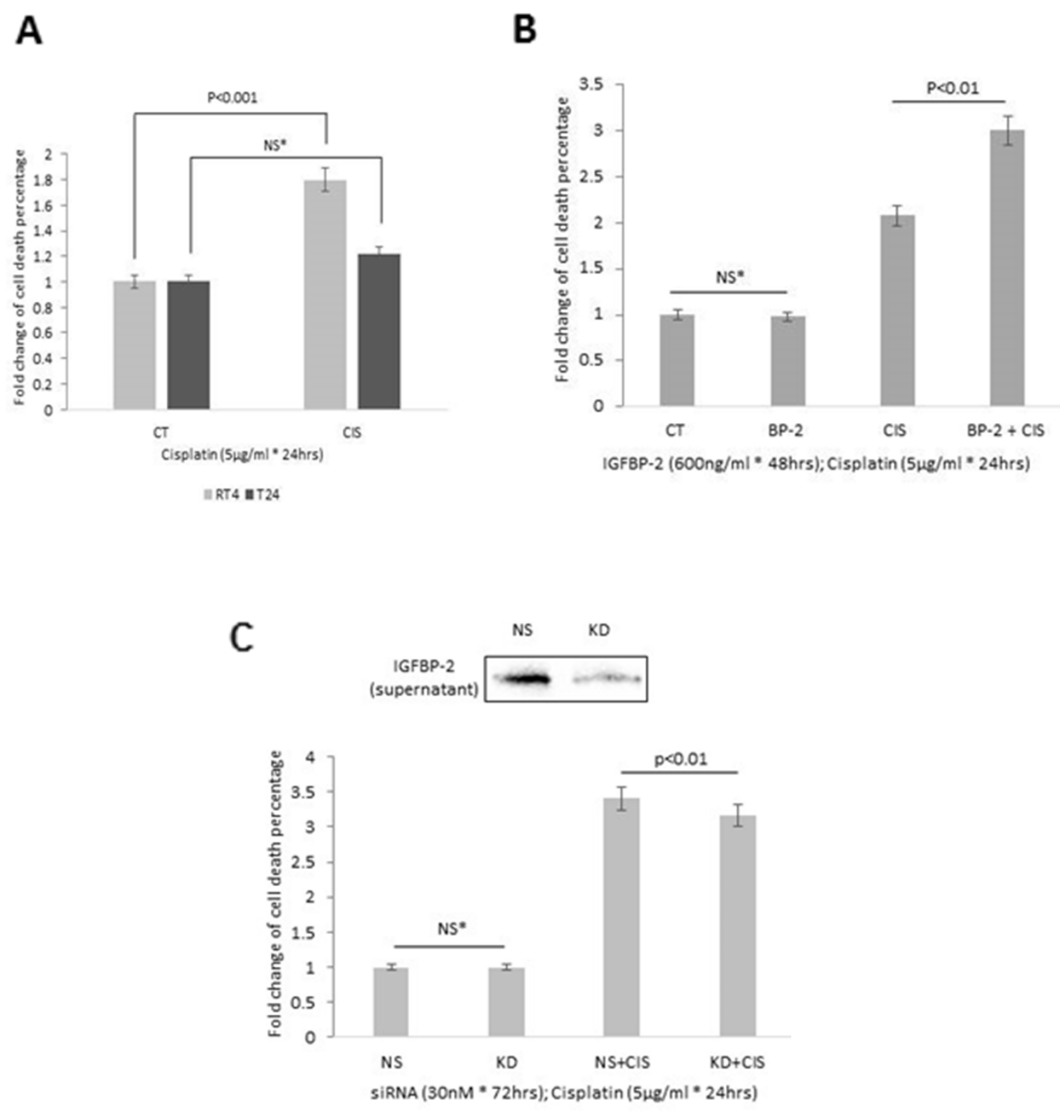
Effects of cisplatin on cell death in **(A)** RT4 and T24 cells and **(B)** in T24 cells following the addition of IGFBP-2 (600ng/ml) and **(C)** in RT4 cells with IGFBP-2 silenced. Insert shows a blot to indicate effective knockdown of IGFBP-2. Graphs show the mean and SEM of data from 3 separate experiments each performed in triplicate. Data were analysed with SPSS 12.0.1 for Windows using one-way ANOVA followed by least significant difference (LSD) post-hoc test. A statistically significant difference was present at p<0.05.

We observed that the epitehlial RT4 cells were more sensitive to cisplatin-induced cell death than the more mesenchymal T24 cells (Figure 6A). As T24 cells do not express IGFBP-2, we added exogenous IGFBP-2 in the presence or absence of cisplatin and found that although IGFBP-2 had no effect on cell death alone it markedly enhanced the sensitivity of the cells to cisplatin (p<0.01; Figure 6B). With RT4 cells, we silenced IGFBP-2 in the presence or absence of cisplatin and found that although silencing IGFBP-2 had no effect on cell death alone, it reduced the sensitivity of the cells to cisplatin (p<0.01; Figure 6C). These preliminary data *in vitro* data suggest that the presence of IGFBP-2 in tumours may play a role in determining chemosensitivity.

## DISCUSSION

Our data suggest that IGFBP-2 acts to inhibit cell proliferation, invasion, colony formation and EMT in bladder cancer and its presence may play a role in determining chemosensitivity. Similar anti-proliferative effects of IGFBP-2 were reported in a human breast cancer cell line, Hs578T [18] and in human embryonic kidney fibroblasts [19]. Whereas opposite results have been reported in MCF7 and T47D breast cancer cells, hepatocellular carcinoma and glioma cells where IGFBP-2 acted to promote cell growth [20, 21]. A reduction of IGFBP-2 was correlated with the promotion of epithelial invasion in the progression of cervical cancer [17], and loss of IGFBP-2 was also found at the invasive front of glioblastoma with high 8, line grade suggesting that IGFBP-2 is associated with a less aggressive phenotype [22]. However, in bladder cancer over-expression of IGFBP-2 enhanced the invasive potential of KoTCC cells. Whilst these data contradict our results and suggest that IGFBP-2 plays a role in inhibiting malignant transformation in bladder cancer, the methodology used to assess the role of IGFBP-2 was very different. As KoTCC cells normally express negligible levels of IGFBP-2, the authors generated an IGFBP-2 KoTCC cell line that over-expressed IGFBP-2. This may not be as physiologically relevant as adding exogenous IGFBP-2 at appropriate concentrations to IGFBP-2 null cells and may therefore not represent the context *in vivo.* Normally IGFBP-2 is a secreted protein and many of its actions are due to interactions with IGFs and with cell-surface proteins/receptors, although some is internalised and may act at critical sites within the cell. In contrast, forced over-expression within the cell may not replicate these intricate interactions but may saturate binding sites within the cell. [16]. In contrast, with certain human leukaemias inhibition of IGFBP-2 resulted in a reduction in cell colony formation activity in a mouse model [23].

A number of changes occur in specific markers during EMT: for example, a reduction in the levels of E-cadherin and an increase in the abundance of N-cadherin. With RT4 cells, the levels of E-cadherin were significantly reduced when IGFBP-2 was efficiently silenced. Changes in RT4 cells indicated that silencing IGFBP-2 promoted a more EMT-like phenotype. While in mesenchymal-like T24 cells, adding exogenous IGFBP-2 led to a significant reduction of N-cadherin expression suggesting inhibition of EMT. These data indicate that the presence of IGFBP-2 may suppress EMT in bladder cancer. As with cadherin switching, in relation to changing from an epithelial to a more mesenchymal cell type, our data appears to suggest that IGFBP-2 is more abundant in epithelial cells, where it is associated with maintaining a more differentiated phenotype. In contrast, mesenchymal bladder cancer cells appear to have negligible levels of IGFBP-2 that correlate with a more aggressive cell type. Again, different conclusions about the role of IGFBP-2 have been drawn in other types of cancers such as pancreatic ductal adenocarcinoma (PDAC), where IGFBP-2 promoted EMT: downregulation of N-cadherin and upregulation of E-cadherin followed knockdown of IGFBP-2 [24]. Its levels were altered depending on the aggressiveness of the cells, being abundant in the less advanced and absent in the most aggressive cells.

Dysregulation of IGFBPs as a result of epigenetic modifications has been identified in many tumours, such as aberrant DNA methylation of the promoter of *IGFBP-2* and *IGFBP-3* genes in hepatomas and breast cancers respectively [25, 26]. We next investigated if altered methylation may be the mechanism by which IGFBP-2 levels were being regulated in bladder cancer cells.

The mesenchymal-like IGFBP-2-negative cell lines, T24 and TCCSUP, were exposed to AZA, a commonly used demethylating agent: re-expression of IGFBP-2 was observed in both cell lines, and the increase in abundance of IGFBP-2 was more significant in T24 cells. In addition to enabling the re-expression of IGFBP-2, treatment with AZA also led to similar phenotypic changes that were observed on addition of exogenous IGFBP-2: with T24 cells, a reduction in abundance of the mesenchymal marker (N-cadherin), significant decreases in cell proliferation and colony formation.

COBRA confirmed that this increase in AZA-induced IGFBP-2 was specifically due to changes in the methylation status of the promoter region of *IGFBP-2* gene in the T24 cells. In TCCSUP cells, as the increase in demethylation in response to AZA was small, we suggest that other epigenetic changes such as histone modification, which has been reported in prostate cancer [27], might also contribute to the loss of IGFBP-2.

Based on the reversible nature of epigenetic modifications, compared with genetic mutations, these findings suggest IGFBP-2 is switched off in advanced bladder cancer which eliminates the inhibitory effects of this protein on cell proliferation, invasion and colony formation in cancer development. In this study, we focussed on methylation but clearly there are other ways in which IGFBP-2 could be modified epigeneically; indeed we showed previously that the levels of IGFBP-2 were enhanced via increased acetylation of histones H3 and H4 associated with the *IGFBP-2* gene promoter [27]. The results of the present study suggest that epigenetic modulation of the *IGFBP-2* gene plays an important part in the loss of IGFBP-2 in progressive mesenchymal bladder cancers, which raises the possibility of epigenetically-targeted cancer therapies for certain bladder cancer subtypes to restore a less aggressive phenotype [28, 29].

As it has been reported that IGFBP-2 can act in either an IGF-dependent or -independent or both manners, NBI-31772, as an inhibitor of IGF/IGFBP-2 interactions, was used to study how it works in bladder cancer. Stimulatory effects of pre-treatment with NBI-31772 alone on both cell growth and invasion of RT4 cells indicated that NBI-31772 freed endogenous IGFs from IGF:IGFBP complexes, mainly IGF:IGFBP-2, to act on the cells to promote growth and invasion alone. This suggested that endogenous IGFBP-2 was acting in an inhibitory manner in part through interactions with IGFs. Silencing IGFBP-2 in RT4 cells also exerted promoting effects on both cell growth and invasion, and also stimulated the same percentage increase in the presence or absence of NBI-31772, with the percentage change being additive to the effects of NBI-31772 lone, which suggested that IGFBP-2 has the ability to act in both an IGF-dependent and -independent manner in epithelial-like bladder cancers. In IGFBP-2-null mesenchymal-like T24 cells, compared with the increase in cell growth and invasion following the addition of exogenous IGF-I, no notable difference was found when cells were treated with NBI-31772 alone, which indicated little endogenous IGFs were released from IGF:IGFBP complexes in these cells. Exogenously added recombinant IGFBP-2 led to a significant reduction both in cell growth and invasion of T24 cells. The inhibitory effect on growth was inhibited by the addition of NBI-31772. This suggested exogenous IGFBP-2 inhibited cell growth by sequestering endogenous IGFs and was no longer able to do this in the presence of NBI-31772, which implied an IGF-dependent action of the exogenously added IGFBP-2 on T24 cell proliferation which has been reported in colon cancer [19]. However, the inhibitory effect of IGFBP-2 on cell invasion was not affected by adding NBI-31772, thus indicating that IGFBP-2 acted in an IGF-independent manner to inhibit invasion of T24 cells.

We observed that the mesenchymal T24 cells are more resistant to cisplatin than the RT4 epithelial-like cells. As T24 cells do not express IGFBP-2, we treated the cells with cisplatin and exogenous IGFBP-2 and found that chemosensitivity was improved. Conversely, silencing IGFBP-2 in the RT4 cells rendered the cells slightly more chemoresistant. A previous study in bladder cancer cells used the BIU87 cell line and established a cisplatin-resistant subline (BIU87-CisR) by continuous exposure of the cells to cisplatin. They found that the the cisplatin resistant subline produced more IGFBP-2 and concluded that IGFBP-2 contributed to chemoresistance [30]. Although seemingly different results the models used are quite different: perhaps upregulation of IGFBP-2 after generating a cisplatin resistant cell line is a compensatory response. In contrast to the sparce data on a possible role for IGFBP-2 in chemosensitivity of bladder cancer, there are convincing data indicating a role for ERCC2 mutations. The abundance of these mutations is reported to be higher in primary compared with secondary muscle invasive bladder cancers (MIBC) and the reduced levels of these mutations in secondary MIBCs correlated with increased chemoresistance [31]. There is also little information about the relative abundance of IGFBP-2 in luminal versus basal bladder cancers. One study did assess mRNA levels of IGFBP-2 and IGFBP-3, and found that whilst a high IGFBP-2 to IGFBP-3 ratio did correlate with a lower recurrence -free survival compared to those with a normal ratio, there were no associations when assessing individual mRNA levels of these proteins [16]. These are interesting observations but a comprehensive study of IGFBP-2 in bladder tumours and its association with chemosensitivity and progression is required to fully understand these effects.

In summary, these data suggest that IGFBP-2 may act as a tumour suppressor in bladder cancer cells as it inhibits cell growth, invasion, colony formation and reduces markers of EMT. Furthermore, methylation may be one mechanism that bladder cancers use to reduce levels of IGFBP-2 to promote carcinogenesis. Our work also provides preliminary data suggesting that the presence of IGFBP-2 in bladder cancer cells may enable them to be more responsive to chemotherapy, but this requires further investigation.

## MATERIALS AND METHODS

### Reagents

Recombinant IGFBP-2 was purchased from GroPep (Thebarton, S.Australia) and 5-aza-2′-deoxycytidine (5-AZA) from Sigma-Aldrich (Dorset, UK). NBI-31772 was purchased from Tocris (Bristol, UK) and is a small non-peptide molecule (molecule weight: 341) which was identified almost two decades ago [6]. With no biologic activity at the IGF receptors, it interacts with IGFBPs by competing specifically against IGF to bind all the six IGFBPs non-selectively with high affinity, and thus displaces free endogenous IGF-I and IGF-II from IGF:IGFBP complexes increasing the free levels of IGF-I and/or IGF-II [6]. The freed IGF-I has been shown to be bioactive in both *in vivo* and *in vitro* studies; therefore, this molecule has been used as a nonspecific IGFBP inhibitor or IGF-potentiator enhancing IGF signalling. Differences in the binding activity of NBI-31772 toward six IGFBPs were identified with the highest being to IGFBP-2 and IGFBP-4, and the lowest to IGFBP-6 [6].

### Cell culture

Human urinary bladder cancer cell lines including RT4, T24, UMUC3 and TCCSUP were purchased from the American Type Culture Collection (ATCC, Manassas, VA). The RT4 cells were cultured in McCoy’s 5A Medium (Modified) (Lonza,) and supplemented with 10% foetal bovine serum (FBS, Fisher Scientific, Loughborough, UK), 1% penicillin and streptomycin (50 IU/ml, Fisher Scientific) and 1% L-glutamine solution (200mM, Sigma-Aldrich). The T24 cells were cultured in Dulbecco’s Modified Eagle’s Medium (DMEM, Sigma-Aldrich), with 4500 mg/L glucose and the UMUC3 and TCCSUP were cultured in Eagle’s Minimum Essential Medium in Earle’s BSS (Fisher Scientific) with non-essential amino acids and supplemented as for the RT4 cells.

### Cell proliferation and cell death

Cell proliferation was assessed using a trypan blue dye exclusion assay as described previously [32, 33].

### Transfection of cells with IGFBP-2 siRNA

Cells were transfected with siRNA using HiPerfect following the manufacturer’s instructions and as described previously [32]. IGFBP-2 was silenced using siRNA (target sequence IGFBP-2; CCCGGAGCAGGTTGCAGACAA; 30 nM for 72 hrs). A non-silencing negative control siRNA was used as a control (30 nM for 72 hrs).

### Cell Invasion

Cell invasion assays were performed as described previously [33]. In brief cells transfected with either non-silencing or IGFBP-2 siRNA (30 nM for 72 hrs) were trypsinized and then seeded into collagen-coated inserts at a density of 0.1×10^6^ cells/insert (RT4) and 0.08 × 10^6^ / insert (T24 and TCCSUP) and allowed to migrate for 24 hours. Membranes were then imaged using a Leica DMI 6000B microscope in 10 random fields (10x magnification). Analysis of the number of invaded cells was performed using Image J (NIH). The supernatant was collected prior to trypsinization to ensure effective silencing of IGFBP-2.

### Subcellular fractionation

Extraction of cytoplasmic and nuclear protein fractions was achieved using a NE-PER Nuclear and Cytoplasmic Extraction Kit (Fisher Scientific). Whole cell fractions were obtained by routine whole cell lysis using cell lysis buffer. Protein concentrations were determined using Pierce BCA Protein Assay (Fisher Scientific) and equal amounts of cytosolic, nuclear and whole cell extracts were analysed by Western blotting.

### Western immunoblotting

Western blot analysis was performed as described previously [32, 33]. Briefly, 20 μg of protein were run on 10% SDS-PAGE , transferred to nitrocellulose membrane and immunoblotted with the following antibodies: fibronectin (1:500, BD Bioscience, CA, USA), E-cadherin (1:1000, Cell Signalling, London, UK), vimentin (1:500, BD Biosciences), FASN (1:1000, BD Bioscience), ERα (1:750, Santa Cruz, Heidelberg, Germany), cyclin D1 (1:1000, Santa Cruz), caveolin-1 (1:500, Santa Cruz), GAPDH (1:5000, Millipore, Watford, UK) and tubulin (1:5000, Millipore), following the manufacturer's instructions. After incubation with specific secondary antibodies conjugated to peroxidise (Sigma-Aldrich), proteins were visualised by Clarity ECL substrate (BioRad, Hertfordshire, UK) using BioRad Chemidoc XRS + system and analysed using Image lab software.

### Colony formation assay

Colony Formation assays were performed as described previously [26]. In brief, cells transfected with either non-silencing or IGFBP-2 siRNA (30 nM for 72 hrs) were trypsinized and then seeded at 10,000 cells/dish. After 3 weeks, the number and the average size of the colonies were counted and calculated using Image J. The supernatant was assessed to ensure efficiency of IGFBP-2 knock-down.

### Combined bisulfite restriction assay (COBRA)

COBRA was used to investigate the methylation status of the *IGFBP-2* gene promoter. DNA from untreated and treated cells was extracted and bisulfite converted with EZ DNA Methylation-DirectKit (ZymoResearch, CA, USA) according to the manufacturer's instructions and as described previously [27]. The primer pair regions in the promoter of *IGFBP2* gene were designed using MethPrimer tool sequences of primers; IGFBP2 forward, 5′-GATTGAAAT-TTATTTGAAGGTTAAAA-3′ and reverse, 5′-ACTCTAAAAATT-CCCTACTCTTCC-3′ and purchased from Thermo Fisher.

### Statistical analysis

Data were analysed with SPSS 12.0.1 for Windows using one-way ANOVA followed by least significant difference (LSD) post-hoc test. A statistically significant difference was present at p<0.05.

## SUPPLEMENTARY MATERIALS FIGURE


