# Supplementary material for: IGFBP-2 acts as a tumour suppressor and plays a role in determining chemosensitivity in bladder cancer cells
Source: Oncotarget. 2019 Dec 17;10(66):7043–57. doi: 10.18632/oncotarget.27355 (PMC6925026; doi:10.18632/oncotarget.27355)
Supplement: Supplementary file 1 [file oncotarget-10-7043-s001.pdf]

IGFBP-2 acts as a tumour suppressor and plays a role in determining chemosensitivity in bladder cancer cells

SUPPLEMENTARY MATERIALS

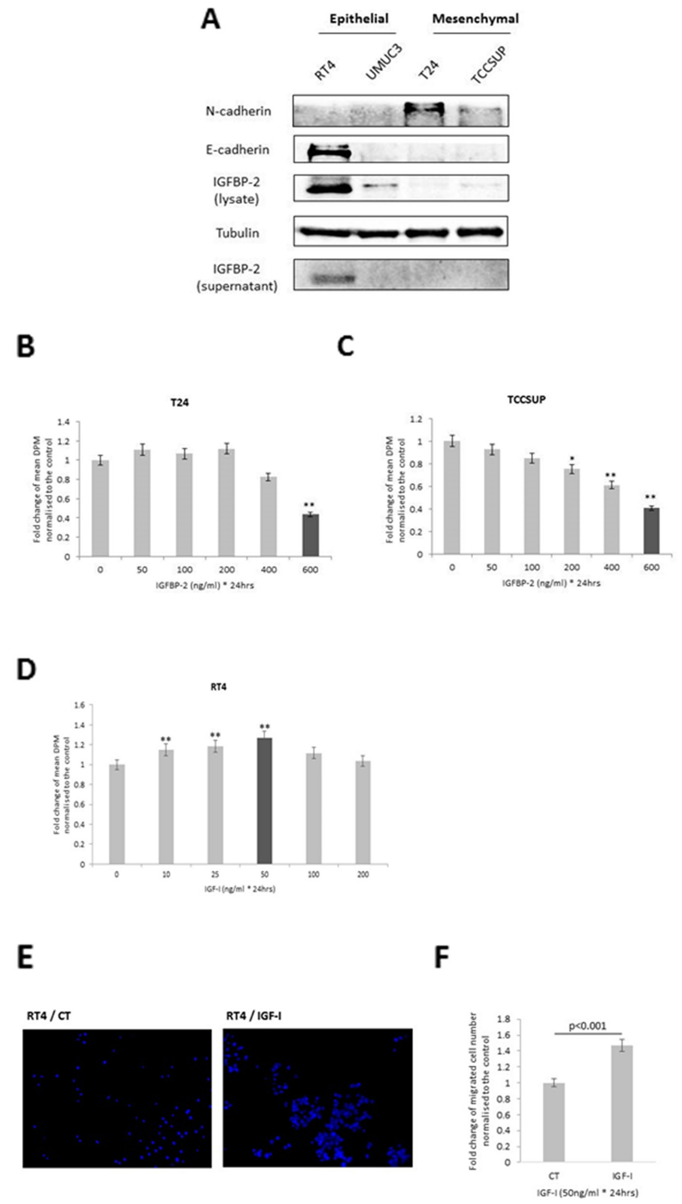

Supplementary Figure 1: (A) Western blots to indicate the levels of E- and N-cadherin and IGFBP-2 in a panel of bladder cancer cell lines, including RT4, UMUC3, T24 and TCCSUP. **(B &C)** Graphs show the effects of IGFBP-2 (0-600 ng/ml) on the proliferation of T24 and TCCSUP respectively. **(D)** Graph shows the effect of IGF-I (0-200 ng/ml) on the proliferation of RT4 cells. **(E & F)** Image shows invaded RT4 cells with the nuclei stained with DAPI (in blue); X 10 magification and the graph indicates the mean change in number of invaded cells. Graphs show the mean and SEM of data from 3 separate experiments each performed in triplicate. Data were analysed with SPSS 12.0.1 for Windows using one-way ANOVA followed by least significant difference (LSD) post-hoc test. A statistically significant difference was present at $p< 0.05$.
